# Does Beta-Trace Protein (BTP) Outperform Cystatin C as a Diagnostic Marker of Acute Kidney Injury Complicating the Early Phase of Acute Pancreatitis?

**DOI:** 10.3390/jcm9010205

**Published:** 2020-01-11

**Authors:** Justyna Wajda, Paulina Dumnicka, Mateusz Sporek, Barbara Maziarz, Witold Kolber, Anna Ząbek-Adamska, Piotr Ceranowicz, Marek Kuźniewski, Beata Kuśnierz-Cabala

**Affiliations:** 1 Department of Anatomy, Jagiellonian University Medical College, 31-034 Krakow, Poland; justynawajda87@tlen.pl (J.W.); msporek1983@gmail.com (M.S.); 2 Department of Medical Diagnostics, Faculty of Pharmacy, Jagiellonian University Medical College, 30-688 Krakow, Poland; 3 Surgery Department, The District Hospital, 34-200 Sucha Beskidzka, Poland; 4 Department of Diagnostics, Chair of Clinical Biochemistry, Faculty of Medicine, Jagiellonian University Medical College, 31-501 Krakow, Poland; mbmaziar@cyf-kr.edu.pl (B.M.); mbkusnie@cyf-kr.edu.pl (B.K.-C.); 5 Department of Surgery, Complex of Health Care Centers in Wadowice, 34-100 Wadowice, Poland; w.kolber@wp.pl; 6 Diagnostics Department of University Hospital in Krakow, 31-501 Krakow, Poland; azabek@su.krakow.pl; 7 Department of Nephrology, Jagiellonian University Medical College, 31-501 Kraków, Poland; marek.kuzniewski@uj.edu.pl

**Keywords:** beta-trace protein, cystatin C, acute pancreatitis, severity, acute kidney injury

## Abstract

Acute pancreatitis (AP) belongs to the commonest acute gastrointestinal conditions requiring hospitalization. Acute kidney injury (AKI) often complicates moderately severe and severe AP, leading to increased mortality. Among the laboratory markers proposed for early diagnosis of AKI, few have been studied in AP, including cystatin C and neutrophil gelatinase-associated lipocalin (NGAL). Beta-trace protein (BTP), a low-molecular-weight glycoprotein proposed as an early marker of decreased glomerular filtration, has never been studied in AP. We investigated the diagnostic usefulness of serum BTP for early diagnosis of AKI complicating AP in comparison to previously studied markers. BTP was measured in serum samples collected over the first three days of hospital stay from 73 adult patients admitted within 24 h of mild to severe AP. Thirteen patients (18%) developed AKI in the early phase of AP. Serum BTP was higher in patients who developed AKI, starting from the first day of hospitalization. Strong correlations were observed between BTP and serum cystatin C but not serum or urine NGAL. On admission, BTP positively correlated with endothelial dysfunction. The diagnostic usefulness of BTP for AKI was similar to cystatin C and lower than NGAL. Increased BTP is an early predictor of AKI complicating AP. However, it does not outperform cystatin C or NGAL.

## 1. Introduction

Beta-trace protein (BTP), also known as lipocalin-type prostaglandin D2 synthase, is a monomeric 168-aminoacid protein belonging to the lipocalin family. Its molecular weight varies according to glycosylation pattern and equals from 23 to 29 kDa [1]. BTP was initially detected in cerebrovascular fluid and was shown to be synthesized in the central nervous system. Currently, BTP serves as a laboratory marker of cerebrovascular fluid leakage, and, for that purpose, robust automated measurement method has been developed and is available in routine laboratories [2,3]. Further studies revealed BTP is expressed in other organs, e.g., heart, lungs or kidneys, and the protein is present in biological fluids such as blood and urine [4]. The low molecular weight allows for free glomerular filtration of BTP present in blood. These characteristics enabled the use of BTP as a laboratory marker of renal filtration [5].

Increased concentrations of BTP in serum or plasma and in urine correlate well with decreased glomerular filtration in patients with chronic kidney disease and the significant increase is observed in the early stages of the disease [5,6,7,8,9]. Moreover, increased BTP has been proposed as a marker of cardiovascular risk in patients with coronary artery disease and those with heart failure [1]. In patients with decompensated heart failure and after acute myocardial infarction, higher serum BTP was associated with long-term mortality [10,11].

Acute pancreatitis (AP) belongs to the most common acute gastrointestinal conditions requiring hospitalization [12]. Although the initial symptoms of acute abdominal pain, nausea and vomiting are serious, in most patients, the disease resolves without complications. Moderately severe or severe AP associated with local and/or systemic complications develops in approximately 20–30% of patients. The systemic complications, i.e., organ failure, including cardiovascular system, lungs or kidneys, may develop both in the early phase of AP (first 7–10 days) or later and lead to mortality in 20–30% of patients [13,14].

Acute kidney injury (AKI) is estimated to affect 7–20% of all in-hospital patients, and it is even more common in surgery and intensive care units [15,16]. The decline of renal function develops over several hours to several days. AKI is a heterogeneous syndrome, often caused by multiple insults [17]. However, in most patients, it is associated with hypoxia affecting renal medulla, caused by constriction or insufficient perfusion of renal arteries, leading to tubular necrosis and decreased glomerular filtration [18]. In severe acute pancreatitis (SAP), AKI is a common complication and may develop either in early phase in result of hypovolemia, systemic inflammation, and endothelial dysfunction, or in the late phase of AP, in association with sepsis [19,20,21]. The mortality of patients with SAP complicated with AP is twice as high as among those without AKI [13,19].

It is commonly recognized that early diagnosis of AKI may allow more efficient treatment and prevent the high mortality. Therefore, significant efforts are undertaken to find a laboratory marker (or a panel of markers) that would allow early diagnosis or prediction of AKI. Although several markers have been proposed [22,23], only few of them are currently available in routine laboratories and can be measured with short (2–3-h) turn-around times required for timely diagnosis. BTP is one of the markers that can be measured by fast automated immunonephelometric method that is available in most medical laboratories and is routinely used to measure such analytes as cystatin C, C-reactive protein, prealbumin or immunoglobulin chains. Moreover, the half-life of BTP in blood has been estimated to be shorter in comparison to other low-molecular-weight proteins [24]—in humans, it is estimated to be 1.2 h [25]. Thus, one may expect that the dynamic changes in renal function may be well reflected by serum BTP. However, studies evaluating BTP as a marker of AKI are scarce, and we were not able to identify any studies on BTP in AP.

The aim of the present study was the assessment of the diagnostic utility of serum BTP concentrations measured with automated immunonephelometric method for the prognosis of AKI in the early phase of AP. The diagnostic utility of serum BTP concentrations were compared with better characterized markers, i.e., serum cystatin C and serum and urine neutrophil gelatinase-associated lipocalin (NGAL).

## 2. Methods

### 2.1. Study Design and Patients

The retrospective study included two cohorts of patients admitted to hospital surgery departments with the diagnosis of AP. The first cohort was recruited at the Surgery Department, District Hospital in Sucha Beskidzka, Poland, between January and December 2014 [26]. The second cohort was recruited in the Department of Surgery, Complex of Health Care Centers in Wadowice, Poland, between March 2014 and December 2015 [27]. In July 2016, the available stored samples of serum collected from the patients were used to measure BTP. The Bioethical Committee of Jagiellonian University, Kraków, Poland (approval no KBET/247/B/2013) and the Bioethical Committee of the Beskidy Medical Chamber, Bielsko-Biała, Poland (2014/02/06/1) gave agreement for patients’ recruitment and the use of patients’ samples for the present study.

In both medical centers, patients were recruited according to following criteria:

- consecutive adult patients admitted to surgery department with symptoms of AP lasting no longer than 24 hours before admission were asked to join the study, and those who signed the informed consent were included in the study;
- the diagnosis of AP was based on revised 2012 Atlanta classification [28], i.e., AP was diagnosed when two of three diagnostic criteria were met, i.e., characteristic abdominal pain, characteristic signs in abdominal imaging (magnetic resonance imaging, contrast-enhanced computed tomography or ultrasonography); serum amylase or lipase exceeding the upper reference limit more than three times;
- patients with chronic pancreatitis, active cancer, or chronic liver diseases (viral hepatitis, liver cirrhosis) were excluded.

The collected demographic and clinical data included: age and sex; comorbidities including ischemic heart disease, diabetes, pulmonary and renal conditions, obesity defined as body mass index (BMI) >30 kg/m^2^; etiology of AP, pancreatic necrosis or pleural effusions present in imaging, development of systemic inflammatory response syndrome (SIRS), transient or persistent organ failure, need for surgery or parenteral nutrition during the hospital stay, length of hospital stay, severity of AP, and outcome (discharge or death).

Based on clinical and laboratory data obtained on day 1 of the study, the bedside index of severity in AP (BISAP) was calculated [29]. The final severity of AP was defined according to the revised 2012 Atlanta classification [28], taking into account the persistent or transient cardiovascular, pulmonary, or renal failure as defined by modified Marshall scoring system (MMSS) [28], the systemic complications (exacerbation of comorbidities), and the local complications.

AKI was defined according to Kidney Disease Improving Global Outcomes (KDIGO) criteria [30] based on increase in serum creatinine of more than 50% or 26.5 µmol/L over 48 h. Renal failure was defined in agreement with MMSS [28] as serum creatinine concentration exceeding 170 µmol/L.

### 2.2. Laboratory Tests

In both centers, patients’ blood samples were collected on admission (study day 1) and on two consecutive days (study days 2 and 3). A part of laboratory tests were performed on the day of collection in the centers recruiting the patients, these included complete blood counts with leukocyte differential counts, routine biochemistry (serum amylase, urea, creatinine, glucose, bilirubin, C-reactive protein), and coagulation tests (citrated plasma D-dimer). Moreover, urine samples were collected from patients treated in the District Hospital in Sucha Beskidzka on the three days of the study and the measurements of NGAL in urine were performed in the center’s laboratory.

Excess serum samples collected on study days 1 to 3 were aliquoted and stored in −80 °C. BTP, cystatin C, soluble fms-like tyrosine kinase-1 (sFlt-1), and angiopoietin-2 were measured in samples from both study centers. Serum NGAL and uromodulin concentrations were only measured in samples collected in the District Hospital in Sucha Beskidzka.

BTP and cystatin C in sera were measured using immunonephelometric method on Nephelometer II analyzer (Siemens Healthcare, Erlangen, Germany). Serum sFlt-1 was measured by electrochemiluminescence immunoassay using the Cobas 8000 analyzer (Roche Diagnostics, Mannheim, Germany). The reference intervals for BTP and cystatin C in serum were <0.70 mg/L and 0.59–1.04 mg/L, respectively. The concentrations of sFlt-1 in healthy subjects were 63–108 pg/mL [31]. The measurements were performed in the Department of Diagnostics, University Hospital in Krakow. Serum angiopoietin 2 and NGAL were measured by enzyme immunoassays using commercially available kits: Quantikine ELISA Human Angiopoietin 2 Immunoassay (R&D Systems, McKinley Place, MN, USA), Human Uromodulin ELISA and Human Lipocalin-2/NGAL ELISA (BioVendor, Brno, Czech Republic). The reference values determined by the manufacturers of the kits were 1.065–8.907 ng/mL for serum angiopoietin 2 and 37.0–501.0 ng/mL for serum uromodulin. The readings were made with an automatic microplate reader Automatic Micro ELISA Reader ELX 808 (BIO-TEK^®^ Instruments Inc., Winooski, VT, USA). The measurements were performed in the Department of Diagnostics, Chair of Clinical Biochemistry, Jagiellonian University Medical College, Kraków, Poland. Urine NGAL concentrations were measured with chemiluminescent microparticle immunoassay on Architect analyzer (Abbott Diagnostics, Lake Forest, IL, USA).

### 2.3. Statistical Analysis

Number of patients and percentage of appropriate group were reported for categories. The contingency tables were analyzed with Pearson’s chi-squared test. Median (lower; upper quartiles) were reported for quantitative variables as most of the variables were non-normally distributed (the Shapiro–Wilk’s test was used to assess normality). The differences between groups were assessed with Mann–Whitney’s test or Kruskal–Wallis’s analysis of variance. Spearman’s rank coefficient was computed for simple correlations. Logistic regression analysis was used to check whether the differences between AKI and non-AKI subjects remain significant after adjustment for the confounders, i.e., age and prediagnosed renal comorbidity. Odds ratios (OR) for unit change were reported with 95% confidence intervals (95% CI). Receiver operating characteristic (ROC) curves were analyzed to compare the diagnostic accuracy of studied markers. The values of area under the ROC curve (AUC) were reported with 95% CI. The AUCs were compared using a method of Hanley et al. [32]. All statistical tests were two-tailed and the *p*-values of <0.05 indicated significant results. Statistica 12 (StatSoft, Tulsa, OK, USA) with Medical Bundle 3.0 (StatSoft, Kraków, Poland) was used for computation.

## 3. Results

Serum samples of 73 patients were available for the measurements of BTP, including 46 patients recruited in the Surgery Department, District Hospital in Sucha Beskidzka, Poland and 27 patients recruited in the Department of Surgery, Complex of Health Care Centers in Wadowice, Poland. In every case (73 patients), at least one sample was available of those collected within the first two days of the study (i.e., first 48 h of hospital stay). Therefore, we decided to report the maximum result of BTP obtained during the 48 h of hospital stay (or the only result, if only one sample from that time period was available) as our baseline measure.

Further, we also analyzed the BTP results obtained on separate study days. There were 65 samples available from day 1, 63 samples from day 2, and 45 samples from day 3. The whole set of samples (collected on days 1 to 3) allowing for the assessment of BTP changes over the study period was available in 33 patients.

Among 73 patients included in the study, 13 (18%) were diagnosed with AKI (Table 1). Patients with AKI were older and suffered from more severe AP, reflected by higher BISAP scores already on the day of admission, more common organ failure throughout the course of AP, a longer hospital stay and higher mortality (Table 1). A history of renal disease was significantly associated with AKI, although the number of patients with preexisting renal conditions was low (Table 1). The patients who developed AKI were characterized by more pronounced laboratory abnormalities during the first two days of hospital stay: lower hematocrit, higher CRP, higher D-dimer, angiopoietin-2 and sFlt-1 as well as higher results of laboratory tests associated with renal function (serum urea, creatinine, cystatin C and BTP, serum and urine NGAL) (Table 1). No difference was observed between AKI and non-AKI patients regarding minimum serum uromodulin (Table 1).

**Figure 1:**
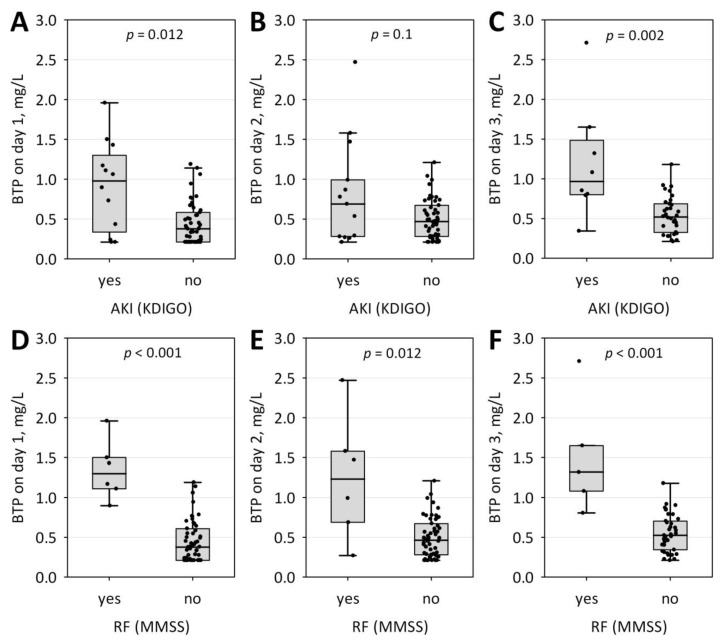
Serum beta-trace protein (BTP) concentrations on day 1 (**A**,**D**), 2 (**B**,**E**) and 3 (**C**,**F**) of hospital stay among patients with AP complicated with acute kidney injury (AKI) diagnosed according to Kidney Disease Improving Global Outcomes (KDIGO) guidelines (**A**–**C**) or renal failure (RF) diagnosed according to modified Marshall scoring system (MMSS) (**D**–**F**) versus patients without these complications. Data are shown as raw data (points), median (central line), interquartile range (box), and non-outlier range (whiskers).

**Figure 2:**
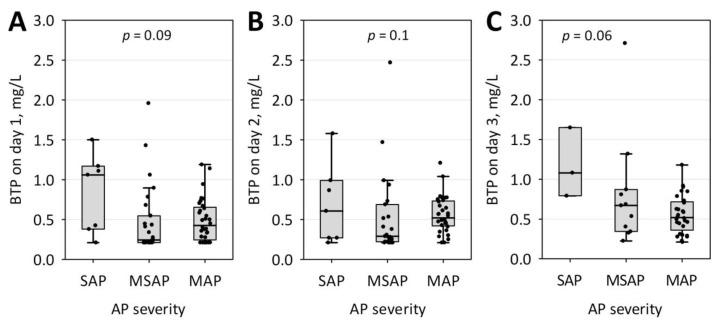
Serum BTP concentrations on day 1 (**A**), 2 (**B**) and 3 (**C**) of hospital stay among patients with acute pancreatitis (AP) of various severity (SAP, severe; MSAP, moderately severe; and MAP, mild) diagnosed according to the modified 2012 Atlanta classification. Data are shown as raw data (points), median (central line), interquartile range (box), and non-outlier range (whiskers).

As shown in Figure 1A–C, serum BTP concentrations on day 1 and day 3 of the study were also significantly higher in patients who developed AKI (*n* = 12 on day 1, *n* = 13 on day 2, and *n* = 8 on day 3) as compared to those who did not. Moreover, BTP concentrations on days 1 to 3 differed significantly between subjects who developed renal failure (diagnosed according to MMSS; *n* = 6 on day 1, *n* = 6 on day 2, and *n* = 5 on day 3) and those who did not (Figure 1D–F). In contrast, serum BTP did not differ significantly between patients with mild, moderately severe and severe AP (Figure 2). Only day 3 BTP concentrations significantly correlated with BISAP score (R = 0.60; *p* < 0.001) and the duration of hospital stay (R = 0.34; *p* = 0.030). No statistically significant changes in BTP concentrations over the three days of the study were observed.

Strong positive correlations were observed over the studied period between serum BTP and other studied laboratory markers increasing in result of impaired renal filtration, i.e., serum creatinine, cystatin C and urea (Table 2). Consequently, negative correlations were observed between serum BTP and serum uromodulin. In contrast, there was no significant correlation between BTP and the marker of tubular injury, i.e., urine NGAL, and the positive correlations between BTP and serum NGAL were not consistent throughout the study (Table 2). BTP measured on day 1 following admission as well as the maximum concentrations recorded on the first two days of hospital stay were also positively correlated with the studied markers of endothelial dysfunction: angiopoietin 2 and sFlt-1 (Table 2). No correlations were observed between BTP and the markers of inflammation: C-reactive protein, white blood cell and neutrophil counts (Table 2).

Serum BTP was highly correlated with patients’ age (R = 0.65 for maximum BTP recorded during first two days of hospital stay; R from 0.65 on day 1 to 0.77 on day 3; *p* < 0.001 for all correlations). In logistic regression, the association between BTP and AKI became statistically insignificant after adjustment for age and preexisting renal pathology (*p* > 0.05 throughout the study), and only BTP concentrations on day 3 of hospital stay were significantly associated with AKI after adjustment for preexisting renal disease only (Table 3). This contrasts with cystatin C that proved a significant predictor of AKI after adjustment for renal comorbidity (Table 3).

**Figure 3:**
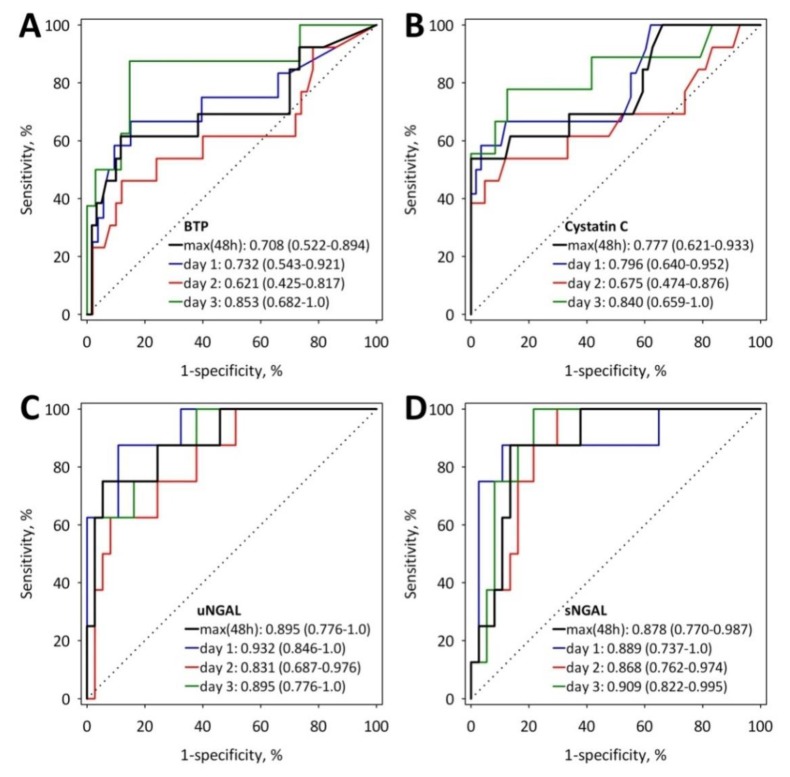
ROC curves showing the diagnostic accuracy of serum BTP (**A**), cystatin C (**B**), urine NGAL (**C**) and serum NGAL (**D**) measured at the specified time-points for the diagnosis of AKI. The values of AUC with 95% CI are shown on the graphs; max(48h), maximum value observed during the first two days (or 48 h) of hospital stay.

In ROC curve analysis, maximum BTP concentrations observed during the first two days of the study as well as BTP concentrations on day 1 and day 3 showed moderate diagnostic accuracy for AKI (AUC from 0.621 to 0.803; Figure 3A). For comparison, we presented the ROC curves for serum cystatin C, serum NGAL and urine NGAL in the diagnosis of AKI (Figure 3B–D). The values of AUC for these markers did not differ significantly from the AUCs of BTP at all the studied time-points (*p* > 0.05 for all comparisons). However, it should be noted that the diagnostic accuracy of serum BTP and cystatin C was very similar while the estimations of AUC for both serum and urine NGAL were consistently higher throughout the study.

## 4. Discussion 

Most laboratory markers proposed for the fast prognosis or diagnosis of AKI have not been studied in AP [19]. Previously, serum cystatin C has been shown to predict AKI in AP with high diagnostic accuracy [33]. Our study shows for the first time that serum BTP, a marker of glomerular filtration, is increased early in patients with AP who develop AKI, in parallel to serum cystatin C. BTP concentrations were highly correlated with serum creatinine and cystatin C. The diagnostic accuracy of BTP for early diagnosis of AKI in the early phase of AP was comparable to that of serum cystatin C. However, it seemed lower than the diagnostic accuracy of serum and urine NGAL. Moreover, in logistic regression analysis, the association between increased BTP and AKI was dependent on previous renal disease, in contrast to what was found for serum cystatin C.

Although creatinine currently remains the main clinically used marker of glomerular filtration, and the only one acknowledged in clinical guidelines for the diagnosis of AKI [30], it is also commonly regarded the late marker of AKI. Significant increase in serum creatinine is not observed in mild renal impairment, in contrast to serum BTP and cystatin C [1,34]. Moreover, there are many non-renal factors associated with altered production of creatinine that affect serum creatinine concentrations, such as muscle mass, age, sex, race, or liver dysfunction [35,36]. Serum cystatin C and BTP seem less affected by non-renal determinants. However, the evidence is mostly based on the studies including patients with chronic kidney disease [35,36]. Of note, serum creatinine production is diminished in liver dysfunction, while BTP concentrations are not affected [37]. This is of importance, as hepatic dysfunction often accompanies SAP. As a marker of glomerular filtration, serum BTP may have an advantage over serum cystatin C in patients treated with glucocorticoids, including those after renal transplantation [1]. On the other hand, recent study that compared serum BTP with serum cystatin C in elderly patients showed better correlation of cystatin C based estimated glomerular filtration rate with measured filtration rate [38].

In AKI, there are very limited data on diagnostic accuracy of BTP. Recently, Saydam et al. [39] evaluated serum BTP, cystatin C and NGAL in comparison with serum creatinine in 57 patients after cardiopulmonary bypass of whom 24 developed AKI. Higher preoperative cystatin C and BTP were associated with AKI, reflecting higher risk for AKI in patients with chronic kidney impairment. Postoperative increase in cystatin C was better predictive marker of AKI than BTP. In our study, serum BTP and cystatin C measured on first three days of AP had similar diagnostic accuracy for AKI (similar areas under the ROC curves). However, the association between BTP and the development of AKI was more affected by kidney disease preceding AP and became insignificant after adjustment for age. Older age is associated with a decrease in glomerular filtration rate, and this decrease results in chronically increased serum concentrations of BTP [38]. Kidney disease preceding the development of AP in our patients could also be a cause of chronically decreased glomerular filtration and increased BTP. We may hypothesize that the increase in BTP associated with the development of AKI is less dynamic in such patients as compared with the increase in cystatin C. However, this interpretation needs to be tested in larger study as the numbers of patients with AKI in our study is low, adversely affecting the power of multiple statistical models, and the number of patients with prediagnosed renal disease in our study is very low.

Serum cystatin C has been evaluated as a marker of AKI in various settings, including sepsis [40,41] and AP [33]. Recent study in over 200 patients with AP reported excellent diagnostic accuracy for AKI (area under ROC curve of 0.948) [33]. In our study, the diagnostic accuracy of cystatin C was lower. However, both SAP and AKI were more prevalent in our study group (9.6% and 18% versus 5% and 7.6%, respectively). Our study included patients admitted within 24 h from the onset of AP symptoms; this time-period was not defined in the study of Chai et al. [33] and might be longer. Moreover, Chai et al. [33] excluded patients with prediagnosed renal disease, whereas in our group four patients had kidney disease diagnosed before the onset of AP.

Serum and urine NGAL are known markers of AKI [23]. In AP, good diagnostic accuracy for AKI of both serum and urine NGAL was previously reported by Siddappa et al. [42] (AUCs of 0.8 to 0.9), comparable with our present results. To our knowledge, there is no published evidence comparing the accuracy of NGAL and BTP in the diagnosis of AKI. Our findings suggest that in AP, the decrease in glomerular filtration reflected by increased BTP is not strictly accompanied by simultaneous increase in NGAL (we did not observe consistent correlations between the markers over the study period). Moreover, the diagnostic accuracy of both serum and urine NGAL for AKI was better than observed for BTP, although it must be remembered that NGAL measurements were available only in patients from one study center.

Cystatin C and BTP are both the low-molecular-weight proteins, easily filtered in renal glomeruli. Increased serum concentrations of both proteins reflect decreased renal function (decreased glomerular filtration rate). Both proteins may be easily measured in serum with automated laboratory methods. However, an international standard is only available for cystatin C, allowing the standardization of the assays [43]. In contrast, the measurements of serum NGAL have not been automated. Moreover, neutrophils are important source of NGAL in serum of patients with acute inflammation, which may decrease the diagnostic accuracy of this marker for AKI as has been shown in septic patients [44]. Urinary NGAL may be measured with an automated laboratory method. However, the sample—urine—may not be available in some patients with AKI. Increased NGAL reflects the injury to proximal and distal renal tubules, irrespective of glomerular function. A combination of a functional marker, i.e., serum BTP or cystatin C with the marker of tubular injury, i.e., serum or preferably urine NGAL might be proposed for the diagnosis of AKI in AP, to be verified in a larger, prospective study.

AP is associated an acute inflammation, which influences the concentrations of many serum proteins. We have not found significant correlations between serum BTP and the inflammatory markers (C-reactive protein, white blood cell and neutrophil count), even though higher CRP was observed in patients with AKI. BTP concentrations did not differ significantly between patients with severe, moderately severe and mild AP. The finding is in line with the evidence regarding patients with chronic kidney disease [25]. However, BTP has been assigned proinflammatory role in allergies and ulcerative colitis and immunomodulatory role has been suggested in bacterial infections [4]. Thus, larger studies are needed to exclude the weak association between serum BTP and acute systemic inflammation.

On day 1 following patients’ admission, we have found significant positive correlations between serum BTP and the markers of endothelial dysfunction or injury, i.e., serum angiopoietin-2 and sFlt-1. Both these endothelial markers have been shown to predict the severity of AP [26,31,45,46] and have increased in patients with kidney injury complicating AP [26] or have correlated with impaired renal function [46]. Angiopoietin-2 has also been shown to predict AKI in other patient populations, e.g., after myocardial infarction [47], after cardiopulmonary bypass [48], with acute respiratory distress syndrome [49], or among patients of intensive care unit [50]. Endothelial injury and vascular leak syndrome are important pathophysiological factors of organ (including kidney) injury and failure in SAP [51]. BTP synthesis is induced in endothelial cells under shear stress. Through prostaglandin D2 synthesis, BTP exerts vasodilating effects [52]. However, the role of BTP in endothelial dysfunction associated with systemic inflammation or sepsis remains to be elucidated.

Our study has several limitations. It was a post-hoc analysis of a small number of patients recruited in two centers. Although the percentage of patients with AKI was within the range previously reported in AP [19], the number of patients with AKI was low, which must be acknowledged as the main limitation of the study. Larger prospective studies are needed to confirm our preliminary results. We measured BTP in available samples stored frozen in −80 °C for a period of one–two years. However, BTP concentrations have been shown to remain stable over long periods in frozen serum samples [53].

In conclusion, our study showed for the first time that serum BTP increases in the early phase of AP in patients who develop AKI in parallel with serum cystatin C and creatinine. Increased BTP is an early predictor of AKI complicating AP.However, its diagnostic accuracy does not seem better as compared to serum cystatin C or serum and urine NGAL. Serum BTP concentrations in the early phase of AP are not affected by the severity of inflammation but correlates with endothelial dysfunction.

## Figures and Tables

**Table 1:** Clinical characteristics of patients with acute pancreatitis (AP) and the maximum laboratory results obtained on first two days (48 h) of hospital stay (or minimum result in case of uromodulin). The quantitative data were presented as median (lower; upper quartile).

Characteristic	AKI (*n* = 13)	No AKI (*n* = 60)	*p*
Age, years	75 (67; 81)	56 (40; 72)	0.003
Male sex, *n* (%)	7 (54)	29 (48)	0.7
Preexisting comorbidities, *n* (%)	11 (85)	38 (63)	0.1
Ischemic heart disease, *n* (%)	6 (46)	16 (27)	0.2
Diabetes, *n* (%)	3 (23)	6 (10)	0.2
Pulmonary diseases, *n* (%)	1 (8)	5 (8)	0.9
Renal diseases, *n* (%)	3 (23)	1 (2)	0.002
BMI >30 kg/m^2^, *n* (%)	0	9 (15)	0.2
AP etiology			
Billiary, *n* (%)	9 (69)	28 (47)	0.3
Alcohol, *n* (%)	1 (8)	14 (23)	
Hyperlipemia, *n* (%)	0	6 (10)	
Other or idiopathic, *n* (%)	3 (23)	12 (20)	
Pancreatic necrosis, *n* (%)	1 (8)	6 (10)	0.8
Pleural effusion, *n* (%)	8 (62)	27 (45)	0.3
SIRS, *n* (%)	8 (62)	23 (38)	0.1
BISAP score at 24 h, points	3 (2; 3)	1 (0; 2)	0.002
BISAP ≥3 points, *n* (%)	8 (62)	7 (12)	<0.001
Organ failure according to MMSS			
Transient, *n* (%)	8 (62)	15 (25)	0.010
Persistent, *n* (%)	4 (31)	3 (5)	0.004
AP severity			
MAP, *n* (%)	1 (8)	37 (62)	<0.001
MSAP, *n* (%)	8 (62)	20 (33)	
SAP, *n* (%)	4 (31)	3 (5)	
Surgery, *n* (%)	0	5 (8)	0.3
Parenteral nutrition, n (%)	2 (15)	3 (5)	0.2
Length of hospital stay, days	11 (8; 25)	7 (5; 11)	0.012
Mortality, *n* (%)	3 (23)	1 (2)	0.002
Amylase, U/L	829 (619; 1526)	1027 (537; 1897)	0.5
Hematocrit, %	37.4 (33.5; 45.2)	43.4 (41.0; 46.9)	0.011
Leukocyte count, ×10^3^/µL	14.8 (12.3; 22.9)	12.1 (9.9; 16.2)	0.1
Neutrophil count, ×10^3^/µL	11.4 (8.6; 19.6)	9.4 (7.5; 12.9)	0.2
CRP, mg/L	258 (182; 313)	104 (49; 229)	0.018
Glucose, mmol/L	8.93 (8.33; 12.25)	7.78 (6.56; 10.11)	0.06
Bilirubin, µmol/L	48.7 (36.4; 81.0)	35.5 (17.8; 65.1)	0.07
Urea, mmol/L	11.68 (6.72; 15.80)	5.83 (4.21; 6.57)	<0.001
Creatinine, µmol/L	120 (95; 207)	71 (61; 85)	<0.001
Cystatin C, mg/L	2.05 (0.84; 2.70)	0.86 (0.69; 1.13)	0.002
BTP, mg/L	0.897 (0.291; 1.470)	0.459 (0.254; 0.631)	0.019
Serum NGAL, ng/mL	313 (275; 489)	142 (101; 232)	<0.001
Urine NGAL, ng/mL	837 (551; 1252)	38 (20; 68)	0.002
Uromodulin, ng/mL	105 (90; 152)	146 (95; 205)	0.2
D-dimer, µg/mL	6.32 (3.82; 15.69)	2.90 (1.33; 4.20)	0.003
Angiopoietin 2, ng/mL	14.48 (5.77; 23.69)	3.25 (2.37; 5.33)	<0.001
sFlt-1, pg/mL	215 (192; 250)	140 (114; 173)	<0.001

AKI, acute kidney injury; AP, acute pancreatitis; BISAP, bedside index of severity in acute pancreatitis; BMI, body mass index; BTP, beta-trace protein; CRP, C-reactive protein; MAP, mild acute pancreatitis; MMSS, modified Marshall scoring system; MSAP, moderately severe acute pancreatitis; *n*, number of patients; NGAL, neutrophil gelatinase-associated lipocalin; SAP, severe acute pancreatitis; SIRS, systemic inflammatory response syndrome; sFlt-1, soluble fms-like tyrosine kinase-1.

**Table 2:** Simple correlations between serum BTP concentrations in the whole studied group of AP patients and other markers of kidney dysfunction, epithelial dysfunction and inflammation measured at the specified time-points.

Variable	Serum BTP Concentrations			
	Maximum of Day 1 and 2* (*n* = 73)	Day 1 (*n* = 65)	Day 2 (*n* = 63)	Day 3 (*n* = 45)
Urea	R = 0.52; *p* < 0.001	R = 0.50; *p* < 0.001	R = 0.41; *p* < 0.001	R = 0.73; *p* < 0.001
Creatinine	R = 0.56; *p* < 0.001	R = 0.63; *p* < 0.001	R = 0.52; *p* < 0.001	R = 0.71; *p* < 0.001
Cystatin C	R = 0.60; *p* < 0.001	R = 0.68; *p* < 0.001	R = 0.63; *p* < 0.001	R = 0.91; *p* < 0.001
Serum NGAL	R = 0.17; *p* = 0.3	R = 0.43; *p* = 0.007	R = 0.22; *p* = 0.2	R = 0.35; *p* = 0.023
Urine NGAL	R = 0.23; *p* = 0.3	R = 0.17; *p* = 0.38	R = 0.30; *p* = 0.1	R = 0.37; *p* = 0.07
Uromodulin	R = −0.42; *p* = 0.004	R = −0.43; *p* = 0.007	R = −0.44; *p* = 0.003	R = −0.33; *p* = 0.037
D-dimer	R = 0.05; *p* = 0.7	R = 0.31; *p* = 0.012	R = −0.07; *p* = 0.6	R = −0.04; *p* = 0.8
Angiopoietin-2	R = 0.26; *p* = 0.045	R = 0.37; *p* = 0.007	R = 0.11; *p* = 0.4	R = 0.22; *p* = 0.2
sFlt-1	R = 0.25; *p* = 0.044	R = 0.34; *p* = 0.011	R = −0.03; *p* = 0.8	no data
Leukocytes	R = 0.11; *p* = 0.4	R = 0.07; *p* = 0.6	R = −0.02; *p* = 0.9	R = 0.05; *p* = 0.8
Neutrophils	R = 0.10; *p* = 0.4	R = −0.01; *p* = 0.9	R = −0.03; *p* = 0.8	R = 0.09; *p* = 0.6
CRP	R = −0.10; *p* = 0.4	R = 0.15; *p* = 0.2	R = −0.19; *p* = 0.1	R = −0.08; *p* = 0.6

* Minimum of day 1 and day 2 concentrations in case of uromodulin.

**Table 3:** Odds ratios for the diagnosis of AKI according to KDIGO obtained in logistic regression adjusted for prediagnosed kidney comorbidity.

	BTP, per 1 mg/L		Cystatin C, per 1 mg/L	
	OR (95% CI)	*p*	OR (95% CI)	*p*
Maximum of day 1 and 2	1.29 (0.72–2.33)	0.4	2.10 (30.1–698)	0.002
Day 1	1.31 (0.72–2.37)	0.4	14.0 (2.34–83.6)	0.003
Day 2	1.15 (0.53–2.50)	0.7	5.12 (1.24–21.2)	0.021
Day 3	125 (2.7–5796)	0.001	33.2 (2.45–451)	0.006

OR, odds ratio; CI, confidence interval.
